# Microparticles for Sustained Growth Factor Delivery in the Regeneration of Critically-Sized Segmental Tibial Bone Defects

**DOI:** 10.3390/ma9040259

**Published:** 2016-03-31

**Authors:** Giles T. S. Kirby, Lisa J. White, Roland Steck, Arne Berner, Kristofor Bogoevski, Omar Qutachi, Brendan Jones, Siamak Saifzadeh, Dietmar W. Hutmacher, Kevin M. Shakesheff, Maria A. Woodruff

**Affiliations:** 1 Institute of Health and Biomedical Innovation (IHBI), Queensland University of Technology (QUT), Brisban, QLD 4006, Australia; giles.kirby@unisa.edu.au (G.T.S.K.); r.steck@qut.edu.au (R.S.), arne.berner@gmx.de (A.B.); k.bogoevski@qut.edu.au (K.B.); brendanjjones@gmail.com (B.J.); siamak.saifzadeh@qut.edu.au (S.S.), dietmar.hutmacher@qut.edu.au (D.W.H.); 2 Department of Trauma Surgery, University of Regensburg, Regensburg 93164, Germany; 3 School of Pharmacy, University Park, The University of Nottingham, University Park, Nottingham NG7 2RD, UK; lisa.white@nottingham.ac.uk (L.J.W.); Omar.Qutachi@nottingham.ac.uk (O.Q.); kevin.shakesheff@nottingham.ac.uk (K.M.S.)

**Keywords:** growth factor, scaffold, bone, repair, regeneration, microparticle, segmental defect

## Abstract

This study trialled the controlled delivery of growth factors within a biodegradable scaffold in a large segmental bone defect model. We hypothesised that co-delivery of vascular endothelial growth factor (VEGF) and platelet derived growth factor (PDGF) followed by bone morphogenetic protein-2 (BMP-2) could be more effective in stimulating bone repair than the delivery of BMP-2 alone. Poly(lactic-co-glycolic acid) (PLGA ) based microparticles were used as a delivery system to achieve a controlled release of growth factors within a medical-grade Polycaprolactone (PCL) scaffold. The scaffolds were assessed in a well-established preclinical ovine tibial segmental defect measuring 3 cm. After six months, mechanical properties and bone tissue regeneration were assessed. Mineralised bone bridging of the defect was enhanced in growth factor treated groups. The inclusion of VEGF and PDGF (with BMP-2) had no significant effect on the amount of bone regeneration at the six-month time point in comparison to BMP-2 alone. However, regions treated with VEGF and PDGF showed increased vascularity. This study demonstrates an effective method for the controlled delivery of therapeutic growth factors *in vivo*, using microparticles.

## 1. Introduction

Most bone fractures do not require intervention and heal spontaneously, stimulated by a choreographed combination of biological signals [1]. Large bone defects often require intervention and are difficult and costly to treat, causing pain and disability. Worst case scenarios may lead to non-union fractures, a chronic and debilitating issue. The clinical gold standard for the treatment of severe bone defects is autologous bone grafting, but limitations to this approach include donor site morbidity, pain, and lack of graft material [2]. The most common site for severe bone loss in humans, resulting in segmental bone defects, is the tibial diaphysis [3].

Autologous bone grafts contain a mixture of cells, mineralised matrix, and signalling molecules. Research within the field of regenerative medicine has focused on seeking out efficacious alternatives in an effort to mitigate the limitations of autologous sourcing. Growth factors (GFs) can be used to impart bioactive properties onto synthetic scaffolds and bone morphogenetic proteins have been successfully used clinically for the treatment of long-bone fractures and to enhance spinal fusion [4,5]. However, to date, clinical approaches using growth factors have not achieved controlled delivery over extended periods [6,7,8]. Higher than physiological levels of growth factor have been used in clinical therapies for bone regeneration and off-label use of these therapies has led to severe clinical complications [9,10,11,12]. A delivery system able to control the release of GFs and deliver a more physiologically relevant dose could negate these safety risks. Furthermore, extended release could increase the efficacy of a growth factor-based treatment concept.

Growth factors have attained regulatory approval for bone repair in certain clinical situations [5,13]. None of the current clinical therapies utilise prolonged release or temporal control over GF release. As a possible result of this, safety concerns have emerged. In 2011, an entire issue of *Spine* explored the evolving safety profile of using rhBMP-2 in the treatment of spinal injuries and reconstructive surgery [14]. It is widely accepted that the clinical complications reported are associated with high doses and off-label use of GF therapies [9]. Collagen sponges are used, onto which a very high dose of reconstituted BMP solution is applied immediately prior to implantation into a bone defect site. OP-1 (Medtronic) and INFUSE (Olympus Bioscience) use up to 3.5 mg BMP-7 and 12 mg BMP-2 respectively [4,5]. While the current clinical use of growth factor therapies is limited it has become clear that having an appropriate localised dose will offer routes towards enhanced safety [9].

Appropriate administration of growth factors is made difficult by their short *in vivo* stability. Accurate half-life values are difficult to obtain and they are often reported as being well below an hour [15,16,17,18]. The high milligram doses of growth factors currently applied clinically could fall below active physiological concentrations within a day. Since multiple administrations of growth factors are unfeasible, a controlled delivery of growth factors is desirable for prolonged therapeutic benefits. Controlled delivery of growth factors from polyesters has previously been reported, however these studies often demonstrate limited controlled release capability [19,20]. We outline a previously published method that aims to mitigate incompete release and protein denaturation while building on the strengths [21,22].

Hydrogels are the most commonly reported delivery system for GFs [23,24] however, hydrogel scaffolds are generally unable to release GFs for extended periods. Polyesters such as poly(D,L-lactide-co-glycolide) (PLGA) used to deliver GFs have been reported in literature as promising alternatives and techniques to combine GFs with polyesters have been reported [25,26,27] but no current methods offer the consistency and high entrapment efficiencies required to take them into the clinic. Polymer microparticles were selected as a GF delivery system because the dry microparticles have a long storage life and could be attached to different types of load bearing scaffolds or even injected directly. PLGA is a copolymer of lactide and glycolide and degradation rates of this polymer can be altered by changing the lactide:glycolide ratio or molecular weight [28,29]; the inclusion of a more hydrophilic component, such as PLGA-PEG(polyethylene glycol)-PLGA, can be used to provide additional control over polymer hydration and degradation rates. This work is an adaptation of previously published methods to improve growth factor delivery by decoupling release kinetics from polymer degradation [22].

Angiogenesis is important during bone regeneration, as the development of new vessels from the host vascular network facilitates nutrient/waste transport and the migration of cells into the site of repair. Vascular endothelial growth factor (VEGF) is a key regulator of angiogenesis [AAAA]. The human fracture haematoma has potent angiogenic activity, thought to be due primarily due to VEGF expression in response to the fracture process [30,31]. Recent research also supports the synergistic relationship between VEGF and Platelet derived growth factor (PDGF) in the generation of stable vasculature [32].

In this investigation in a well-characterised and validated large preclinical animal model, we studied an implant consisting of two elements: a biodegradable structurally supportive, yet highly porous scaffold made from polycaprolactone (PCL) which was combined with PLGA-based microparticles delivering therapeutic GFs immobilised in place with platelet rich plasma. The treatment of load bearing bones, such as the tibia, requires a mechanically supportive scaffold in combination with an internal fixation device to provide a structure with appropriate mechanical characteristic to support the growing bone matrix.

This research was based on the hypothesis that the combined delivery of VEGF, PDGF and BMP-2 would generate more efficient bone regeneration than BMP-2 alone based on the rationale that this more closely mimics biological events. The angiogenic factors should drive early vasculature and the BMP-2 should drive bone formation. To test this we used a previously reported PLGA microparticle delivery system [21,22] to deliver VEGF and PDGF at an early stage and deliver bone morphogenetic protein 2 (BMP-2) at a later stage. This was compared to the controlled delivery of BMP-2 alone; GF-free microparticles were used as a control.

## 2. Results

### 2.1. Growth Factor Release in vitro

**Figure 1:**
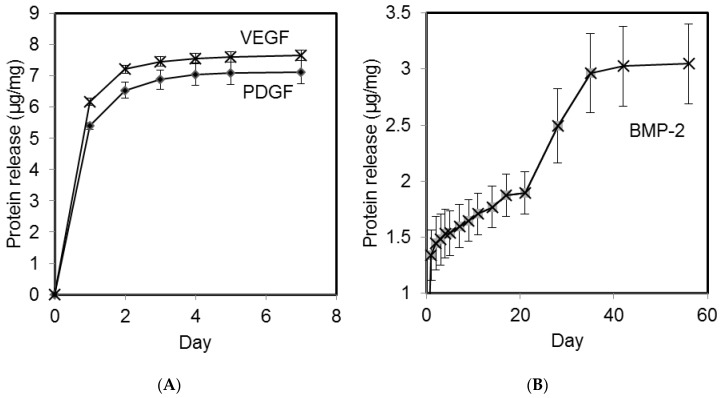
Cumulative protein release per mg of polymer microparticles. Release of VEGF and PDGF from polymeric microparticles occurred over the first five days (**A**); in contrast BMP-2 was released for 35 days (**B**).

Microparticles containing the angiogenic GFs VEGF and PDGF exhibited release over the first 5 days following an initial burst release (Figure 1A) with the PDGF releasing slightly after the VEGF. Microparticles containing BMP-2 exhibited an initial burst release followed a sustained delivery of BMP-2 for 35 consecutive days (Figure 1B). The chosen microparticle formulations demonstrated the ability to impart different durations of release on the angiogenic and the osteogenic growth factors. Neither formulation delivered all the GF that was loaded but this is a well known limitation of GF release formulations [33,34]. All loadings within animals were based on measured GF delivery *in vitro*. A theoretical 100% release would have been 10 µg/mg microparticles. It can be seen that the fast releasing formulation is close to this at approximately 70% whereas the slower formulation is lower at around 30% (Figure 1A and B respectively).

**Figure 2:**
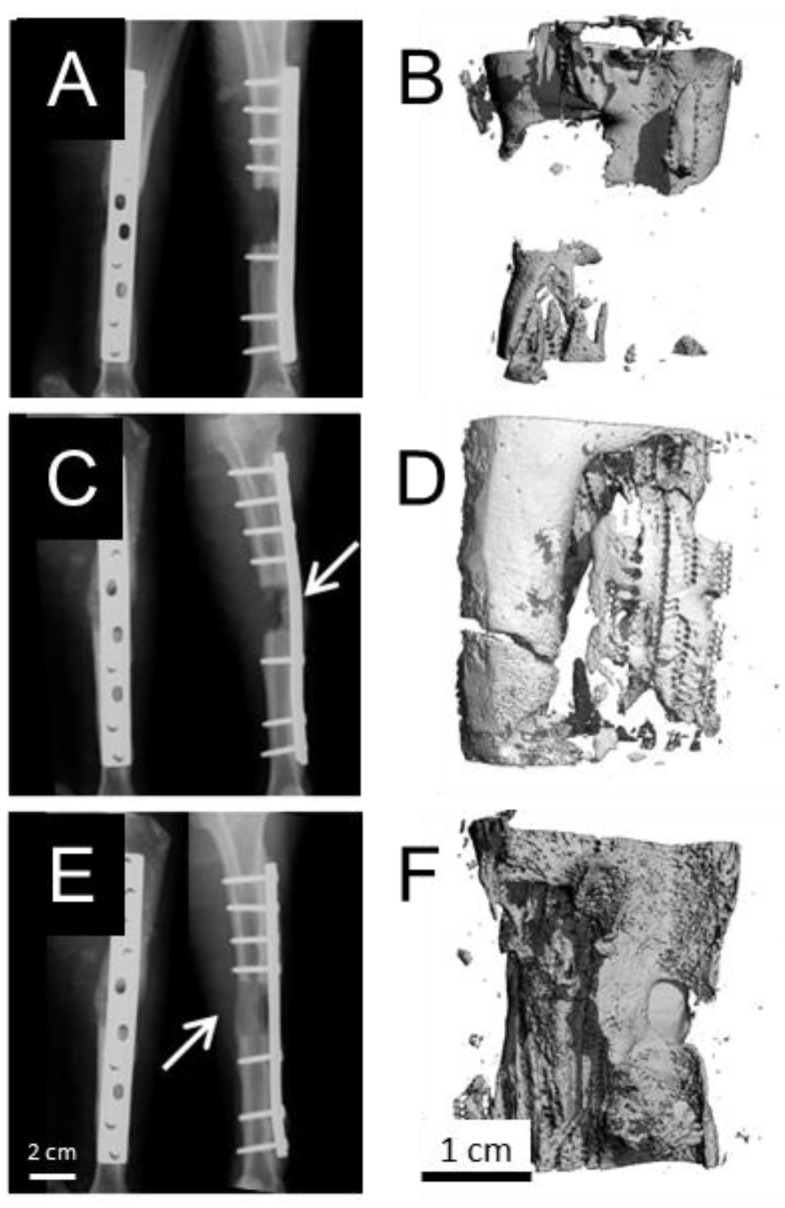
Representative X-rays and micro CT reconstructions of the implant at 6 months indicated that without growth factor inclusion, the defects failed to bridge (**A**); and (**B**). An increased incidence of bridging was observed in groups administered with VEGF, PDGF and BMP-2 (**C**;**D**), as well as groups administered with BMP-2 alone (**E**;**F**).

X-rays of the treated tibiae post operatively confirmed implant stability. X-rays at the 6 month end-point indicated bridging had occurred within 4 of the BMP-2 treated defects, 3 of the combination GF treated defects and just one of the control defects. Mineral density in all explants appeared to be lower than that of native cortical bone. Representative X-rays of the experimental groups are shown (Figure 2). These groups are detailed in Table 1.

### 2.2. Explant Analysis

Defect regions were explanted from the sheep with both proximal and distal native bone. Dynamic compression plates were removed and each sample subjected to mechanical testing at the same time and soon after euthanasia to ensure the results were as consistent and biologically relevant as possible. Each sample was then scanned with micro computed tomography to provide quantitative data prior to fixation and histological analysis. Every sample was subjected to the histological analysis method. To do this, the bone was first sectioned longitudionally. One half was fixed and resin embedded for sectioning of mineralised bone and the other half was cut laterally into smaller parts for decalcification and conventional paraffin histology and immunohistochemistry.

### 2.3. Biomechanical Testing

Three and four defects from the multiple growth factor group and the BMP-2 group, respectively, provided enough resistance under load to generate meaningful mechanical data. The controls had insufficient mechanical strength to be tested; they failed before any strain could be measured. This was due to non-union of the bone.

**Figure 3:**
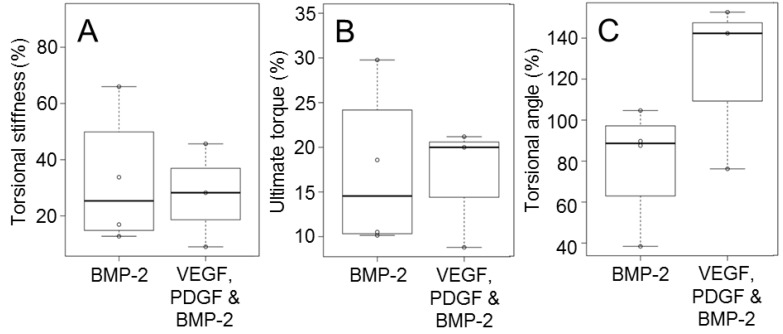
Tibial explants were mechanically tested and normalised against the contralateral leg. This provided the stiffness (**A**); maximum torque (**B**); and the angle of failure (**C**). Boxes indicate one standard deviation above and below the mean.

The GF-free group was excluded from statistical comparisons due to the lack of mechanical data. Statistical comparisons between the multiple growth factor and BMP-2 groups (Table 1) indicated no significant differences with respect to ultimate torque and torsional stiffness (*p*-value = 0.59 and *p*-value = 0.61 respectively). The torsion angle at failure indicated higher average values for the multiple GF group but no significant difference between groups was evident (Figure 3). There was no differences between GF treated groups but GF-free controls failed to bridge at all.

### 2.4. Micro Computed Tomography

The micro CT analysis provided quantitative support for the qualitative conclusions drawn from the clinical X-rays. It support the X-ray result that just one of the five GF-free defects showed defect bridging in contrast to 7 of the 10 GF treated defects (4 of the BMP-2 treated defects, 3 of the combination GF treated defects).

**Figure 4:**
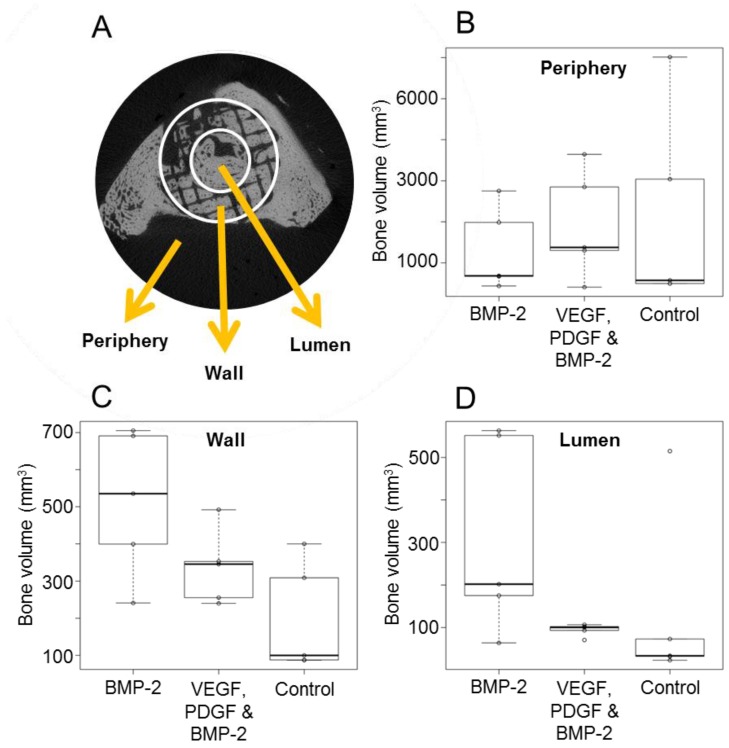
Bone volumes within different defect regions (**A**) were quantified; bone volumes within the periphery (**B**); scaffold wall (**C**); and scaffold lumen (**D**) for each experimental group are shown.

Within the scaffold wall (Figure 4A), both growth factor treated groups demonstrated a higher bone volume than the scaffold only group. The group treated with BMP-2 alone showed the greatest increase in bone. This same trend is seen within the scaffold lumen (inside the scaffold) (Figure 4B). The mean bone volumes inside the scaffold within the growth factor treated groups were, on average, higher than that of the GF-free group. 

All defects exhibited some bone growth in the periphery region (outside the scaffold) ranging from 409 mm^3^ to 6013 mm^3^ (Figure 4B) but there were no significant differences between groups.

### 2.5. Histology

Visual inspection of the explants (prior to embedding) indicated that the PLGA based microparticles had completely degraded whereas the PCL scaffold remained in place providing structural stability. Once the tissue was processed through xylene, the remaining PCL was dissolved. Mechanical testing was carried out before histological processing and deformations in the tissue/scaffold are attributed to this destructive mechanical testing.

**Figure 5:**
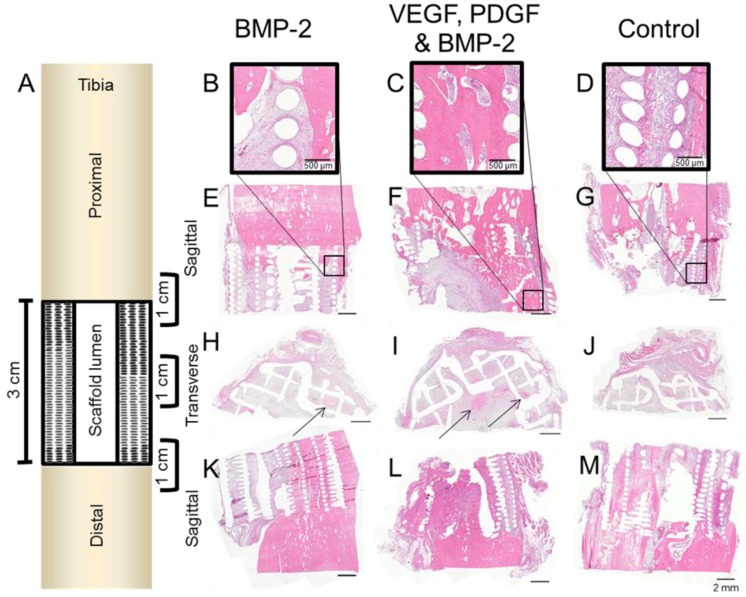
A schematic of the tibial defect containing the scaffold is shown (**A**). Three 1-cm regions were cut and decalcified to show the integration between scaffold and native bone (proximally and distally) and also the mid regions. These regions were sectioned sagittally (**B**–**G**) and (**K**–**M**) and transversely (**H**–**J**).

Representative haematoxylin and eosin (H&E) images from decalcified samples within each experimental group demonstrated good integration between the scaffold and the host bone both proximally and distally (Figure 5). All the regions that contained PCL scaffold appear as empty circles or lines which were once the scaffold struts and had dissolved. Central defect sections cut laterally indicated bone development deep within the scaffold (Figure 5G–I).

**Figure 6:**
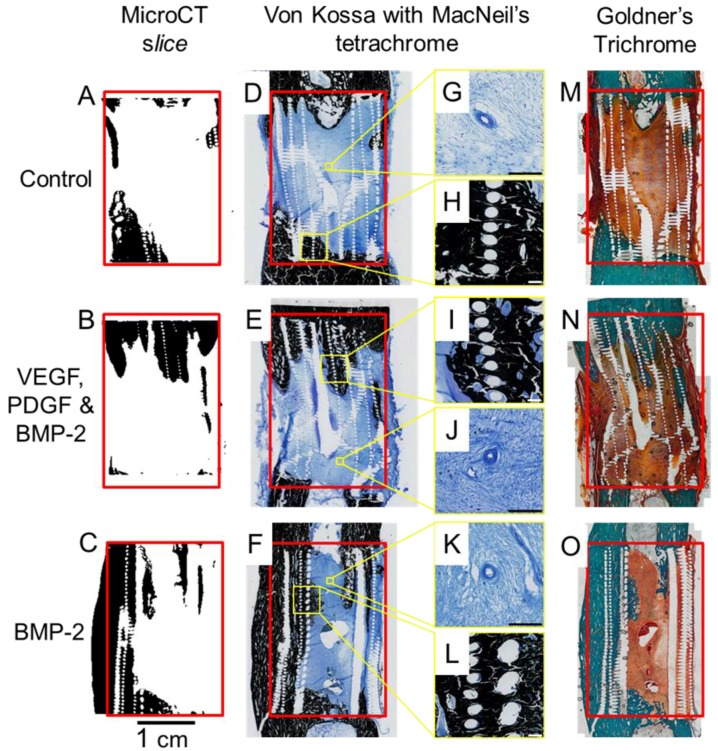
Micro CT slices (**A**–**C**) shown with Von Kossa stained (**D**–**F**) and Goldner’s Trichrome stained (**M**–**O**) counterparts. More bone growth into the scaffold was observed in growth factor treated groups. Magnified regions show both the bone ingrowth and vascular formations (**G**–**L**).

Entire slices (proximal and distal bone plus the scaffold/defect) from each experimental group are shown (Figure 6). Calcified sections (embedded in resin) were stained using Von Kossa/MacNeal’s Tetrachrome and Goldners trichrome to identify mineral deposition and cellular detail. All groups exhibited some degree of bone ingrowth into the scaffold from both the proximal and distal ends. GF treated groups showed bone growth deeper within the scaffold. Sections cut at an appropriate plane verified the mineralised defect bridging seen in the micro CT reconstructions (Figure 6). The Von Kossa and Goldner’s Trichrome results concur with the micro CT and clinical X-ray data. Corresponding micro CT reconstructed sections were compared with the corresponding histology sections (Figure 6). This validated both the micro CT threshold levels and the histological staining. Direct integration between the scaffold and bone can be seen in the Von Kossa stained magnified regions of Figure 6. Blood vessels were also evident in the soft tissue of all groups, these are detailed in Figure 6G,J,K.

The Goldner’s Trichrome staining technique was used to provide a macroscopic overview and compliment the Von Kossa results. The Goldner’s staining results support the micro CT and Von Kossa data with respect to bone and soft tissue distribution within the defects.

**Figure 7:**
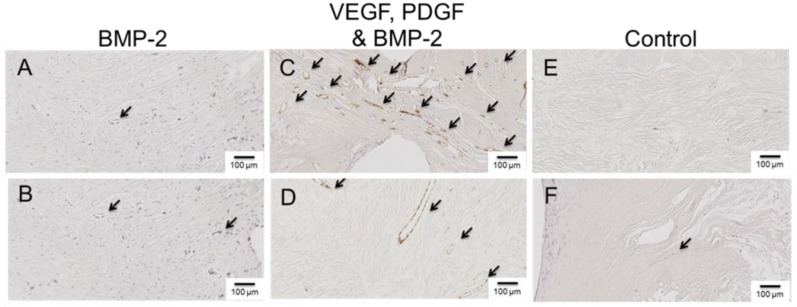
Representative images from defect mid sections showing Immunohistochemical staining for the endothelial marker Von Willebrand Factor (vWF). Defect regions treated with controlled release BMP-2 (**A**–**B**); defect regions treated with controlled release VEGF, PDGF and BMP-2 (**C**–**D**); control defects treated without the inclusion of growth factor (**E**–**F**).

Several antibodies were selected to evaluate the newly formed bone tissue and identify any differences between groups. Von Willebrand Factor (vWF) is a blood glycoprotein involved in hemostasis and by identifying regions of vWF using immunohistochemistry (IHC), we were able to identify blood vessels within the defect/scaffold. The mid-region (sectioned laterally) indicated a difference between groups. In the GF-free and BMP-2 only groups, very few blood vessels could be identified, whereas the VEGF, PDGF and BMP-2 group had a higher density of blood vessels, both large and small (Figure 7).

Osteocalcin, CD68, and Collagen 1 are markers associated with bone growth and these were present within the mineralised regions of all samples (Figure S1). There were no differences between groups.

## 3. Discussion

More bridging was anticipated for GF treated groups but although the high spread of data makes definitive conclusions difficult, the mechanical testing data chearly indicated that the GF treated samples exhibited greater mechanical integrity. An optimum dosing of controlled release GFs remains to be determined but we have demonstrated the successful sustained delivery of GFs to defect sites. This administration regime still has enormous potential to increase both safety and efficacy of GF delivery as developments continue.

Callus formation and neovascularisation into the repair site is known to be important in facilitating the recruitment and distribution of stem cells and osteoblasts in the repair site [31]. VEGF was not expected to have direct effects on osteoblasts but to enhance early vascularisation leading to an increased vascular network with vascular permeability [35,36]. Both VEGF and PDGF were selected because recent studies have suggested the importance of this combination in the generation of a clinically relevant vasculature network [32]. Research into the molecular aspects of bone formation has suggested an important functional link between VEGF-mediated angiogenesis and osteogenesis [37]. It, henceforth, follows that VEGF and potentially other angiogenic growth factors could become important components of future therapies developed towards orthopedic applications. Previous studies investigating the co-administration of VEGF with BMP-2 vary in design and few explore the the function and quality of bone deposited in this way.

While VEGF is important for angiogenesis, there are concerns that prolonged exposure may lead to aberrant effects [38]. It has been shown that co-delivery of VEGF with PDGF results in more stable vasculature [32] and there is thought to be an association between VEGF and BMP-2 during bone formation [39]. Future studies would benefit from the analysis of VEGF and PDGF, individually and together, to fully determine this synergistic relationship in bone repair. In this study, it was unclear whether early angiogenesis enhanced bridging but it is however evident that VEGF and PDGF enhanced vasculature and that controlled release BMP-2 delivery enhanced bridging. Future studies should explore additional time points to fully assess the temporal effects of growth factor relivery.

The ovine tibial critical size defect model is well characterized for bone tissue engineering applciations. It has previously been shown that a scaffold by itself in combination with a fixation plate will not result in clinically predicatble bone volumes for bridging the defct [40], furthermore it has been shown that the addition of platelet rich plasma (PRP) does not enhance bridging above that of a scaffold only [41]. This is the most challenging segmental bone defect model described in the literature for any regenerative approach strategy. Any repair and bridging of such a defect is a strong indication of effectiveness of the treatment.

Controlled release of the administered GFs was a novel aspect of this approach, this was also one of the most challenging aspects. Determining a sustained release therapeutic GF dose was an estimation based on physiological concentrations. The physiological concentration of BMP-2 in bone can be calculated as approximately 0.02 ng/mm^3^ [42] and this is sufficient for bone healing in normal fractures. Our aim was to use a total GF dose lower than current clinical approaches, which are very high (3.5 and 12 mg). The literature relating to VEGF and PDGF dosing in controlled release systems is sparse and administration values were estimated using BMP-2 levels as a benchmark. It has been shown that excessive VEGF can actually inhibit osteogenesis [43] so the total dose of VEGF was fixed to 50% that of BMP-2. The chosen formulations released approximately 2 ng/mm^3^/day BMP-2 and 1 ng/mm^3^/day VEGF and 1 ng/mm^3^/day PDGF.

The polymer formulations selected for this study were successful in staggering the release of growth factors. Both VEGF and PDGF were released at an early stage (up to day 5) and a later-release of BMP-2 was achieved (Days 25–35). Due to the dense way the microparticles were packed within the scaffold lumen (Figure S2), we postulated that the actual growth factor release times may be extended due to non-sink release conditions retarding the passive diffusion of GF from the microparticles. Microparticles loaded with BMP-2 released 30% of the growth factor *in vitro* and issues of incomplete release have been previously reported [44] and widely discussed; reviews have explored the subject in depth [34]. We postulate that in this case, that interactions between GF and polymer led to adsorption. Release data from the microparticles was used to determine the mass of microparticles required to administer to the defect site to deliver an appropriate amount of GF.

It was apparent from histological mineral staining that inclusion of controlled release GFs increased the depth of bone ingrowth into the scaffolds and increased the incidence of bridging (4/5 of the BMP-2 treated defects; 3/5 of the combination GF treated defects). Enhanced bridging was also evident from mechanical testing with bridging localised within the scaffold region. This provided a physiological appearance to the regenerated bone. Localised osteogenesis was an indicator that the system provided a localised region of activity. Mechanical testing showed that the defects treated with VEGF, PDGF and BMP-2 had a greater torsional range than the BMP-2 alone. The cause of this torsional range remains unclear. A greater sample size would have been necessary to begin to draw statistically valid conclusions as the treated groups exhibited less regeneration that was anticipated.

The inclusion of microparticles delivering a controlled dose of BMP-2 was able to increase the bridging of defects in contrast to a GF free scaffold. The addition of VEGF and PDGF to the BMP-2 made no significant improvement over the administration of BMP-2 alone in this ovine critical defect model at six-months.

## 4. Materials and Methods 

Unless otherwise stated, reagents and consumables were purchased from Sigma-Aldrich (Castle Hill, Australia).

### 4.1. Triblock Copolymer Fabrication

Poly(lactic-co-glycolic acid) (PLGA) was supplemented with triblock copolymers consisting of PLGA and polyethylene glycol (PEG) to tailor protein release. PLGA-PEG-PLGA was prepared and analysed using previously described methods [22]. Triblock characteristics are detailed in Table 2. D,L-Lactide and glycolide monomers were obtained from Lancaster Synthesis, Ward Hill, MA, USA and PURAC, Gorinchem, Netherlands, respectively. The synthesis involved a ring opening polymerisation of D,L-lactide and glycolide in the presence of PEG with a small amount of stannous octoate. This method was reported by Zentner *et al.* [45] and adapted by Hou *et al.* [46].

### 4.2. Microparticle Fabrication

Microparticles were prepared according to a previously described method involving a *water/oil/water* double emulsion solvent evaporation technique [21,22]. PLGA polymers with lactide:glycolide ratios of 50:50 and 85:15 (PLGA 85:15 DLG 4A 56 kDa and PLGA 50:50 DLG 4.5A 59 kDa) were purchased from Surmodics (Eden Prairie, MN USA). Briefly, PLGA/triblock (1 g) was dissolved in dichloromethane (5 mL), this formed the *oil* phase. A protein mixture consisting of 1 mg growth factor and 9 mg albumin (from human serum) (HSA), or 10 mg HSA for GF-free microparticles, was dissolved in deionised water (100 µL); this was added to the oil phase. This mixture was homogenised using an LM5 axial impeller mixer (Silverson machines, Ltd, Chesham, UK) for 2 min at 4000 rpm. This primary *water/oil* emulsion was quickly added to a 200 mL bath of polyvinyl alcohol (0.3%) and homogenised again for 2 min at 2000 rpm. This *water/oil/water* emulsion was stirred at 300 rpm for 4 h and the microparticles were then washed and collected before freeze drying. The finished microparticles were free-flowing and of a homogeneous consistency and a mean diameter of 100 µm.

Three different polymer formulations were selected, one for each growth factor. Two variables were utilised to control the rate of growth factor release: the ratio of lactide to glycolide in the PLGA and the weight percentage of PLGA-PEG-PLGA added to the PLGA. The three formulations are detailed in Table 3. The aim was to stage the release of the three growth factors as VEGF followed closely by PDGF followed by BMP-2.

Recombinant human vascular endothelial growth factor (rhVEGF_165_) and recombinant human platelet derived growth factor (rhPDGF-BB) were purchased from PeproTech (London, UK). Recombinant human bone morphogenetic protein-2 (rhBMP-2) was purchased from Professor Walter Sebald (University of Wurzbürg, Wurzbürg, Germany). The microparticles that contained HSA and no growth factor are referred to throughout as GF-free.

An *in vitro* release study was carried out to determine the release rates of the growth factors from different polymer formulations.The release of protein over time was quantified in simulated *in vivo* conditions. Release studies were continued until all the protein was released or the microparticles were fully degraded.

Aliquots (50 mg) of the microparticles (triplicate samples from each batch) were suspended in 1.5 mL phosphate buffered saline (PBS; pH = 7.4); samples were gently rocked in a humidified incubator at 37 °C. At defined time intervals the entire amount of PBS was removed from the 1.5 mL microcentrifuge tubes and replaced with 1.5 mL fresh PBS; all liquid above the microparticles was collected without removing the microparticles. The collected supernatants were stored frozen until required and then assayed using a Micro BCA assay kit (Thermo Scientific, Paisley, UK) with a standard curve consisting of human serum albumin (0–40 µg/mL).

### 4.3. Scaffold Design and Fabrication

Bioresorbable cylindrical scaffolds of medical grade poly-caprolactone (mPCL), (outer diameter: 20 mm, height: 30 mm, inner diameter: 8 mm) were produced by melt extrusion using a BioExtruder (designed by team at the Centre for Rapid and Sustainable Product Development, Leiria, Portugal) [47].

The structural parameters of the scaffolds were tailored by computer-aided design and included specifications of 70% porosity with 100% pore interconnectivity within a pore size of 350–500 μm size and a 0/90° lay-down pattern (Figure 1A). This architectural layout was deemed particularly suitable for load bearing tissue engineering applications since the fully interconnected network of scaffold fibres could withstand early physiological and mechanical stress in a manner similar to cancellous bone [41]. 

Prior to surgery, all scaffolds were surface treated for six hours with 1M sodium hydroxide (NaOH) and washed five times with phosphate-buffered saline (PBS) to render the scaffold more hydrophilic. Scaffold sterilization was achieved by incubation in 70% ethanol (followed by complete evaporation) and UV irradiation for 90 min, rotating the scaffold 3 times.

### 4.4. Loading of Microparticles into the Scaffold

**Figure 8:**
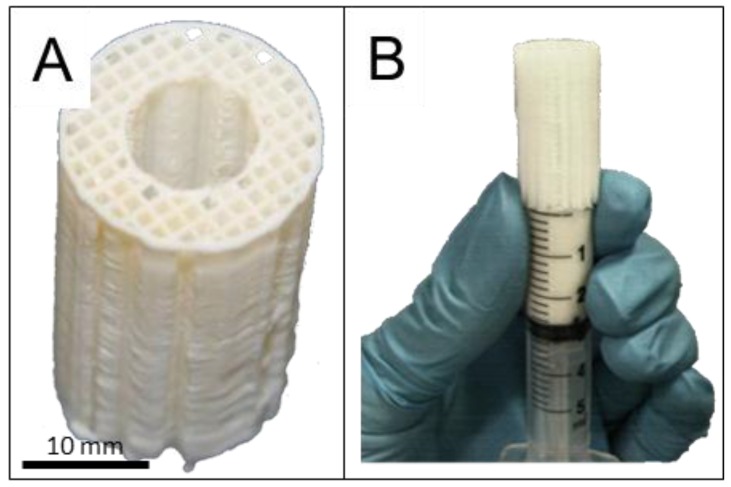
Microparticles were incorporated into a polycaprolactone scaffold which is designed with large pores (>500 micrometers) and fully interconnected honey comb pore architecture (**A**); the microparticle/platelet rich plasma mixture was injected into the scaffold from one end (**B**).

The microparticles were combined with the scaffold to provide a bioactive GF-containing element to the structurally supportive mPCL scaffold as shown in Figure 8. The mixture of microparticles consisted of the same ratio of formulations for each defect and the GF was simply replaced with an equal loading of albumin. This kept the polymer ratios consistent between groups. The microparticles were added into the mPCL scaffold architecture by mixing the microparticles within autologous platelet rich plasma [41] as previously reported. The microparticles were mainly contained in the central region (lumen) of the scaffold thereby maintaining a high level of porosity in the scaffold wall (Figure S2). The microparticles were too large to migrate laterally out from the scaffold and the host tibia essentially blocked the ends. This reduced the likelihood of microparticles migrating from the defect area.

To produce platelet rich plasma (PRP) 80 mL of blood was collected from the jugular vein of each sheep and transferred into 3.5 mL monovettes supplemented with sodium citrate (3.8%) at a ratio of 9 volumes blood and 1 volume sodium citrate according to procedures reported by Anitua *et al.* [48]. The citrated blood was transferred to falcon tubes and centrifuged in a standard laboratory centrifuge for 20 min at 2400 rpm. Subsequently, the yellow plasma layer from all tubes (from a single sheep) was transferred to a fresh falcon tube and the platelets were pelleted in a second centrifugation step for 10 min at 3600 rpm [49]. The pellet was resuspended in 1.2 mL of plasma and the platelets counted in a haemocytometer.

Each sheep was implanted with the same mixture of microparticle formulations, the only difference being whether the microparticles contained growth factor or not. The microparticles (2000 mg, detailed in Table 1) were mixed with 1100 µL PRP. This mixture was injected into the lumen of the scaffold using a modified 5 mL syringe (Figure 8 B). PRP was activated with Thrombin (5 U/mL), which effectively clotted the microparticles within the scaffold pores and the scaffolds were incubated at 37 °C for one hour before implantation into the defect.

### 4.5. Implantation

Ovine tibial resections at the diaphysis provide an excellent preclinical test environment for bone regenerative therapies. A resection of a 3 cm segment is widely accepted to create a non-healing environment and this model has ben standardised and validated [2,40,41,50,51]. At 6–7 years old, sheep undergo secondary osteonal remodelling and thus their bone density and microstructure are comparable to that of humans [50]. Sheep also have an equivalent weight to humans and the dimensions of their long bones are suitably long to allow the use of human implants [52]. This study utilised an established and well-tested version of the ovine tibial resection model carried out at the Medical Engineering Research Facility (MERF), QUT. 

Fifteen mature merino sheep (weight 47 ± 6 kg, aged 6–7 years) were operated upon as approved by the University Animal Ethics Committee of the Queensland University of Technology, Brisbane, Australia (approval number 0900000906). The experimental groups consisted of a scaffold group containing GF-free microparticles (no growth factor, HSA only) which served as a negative control, plus two growth factor containing groups as detailed in Table 1. Power analysis of a previous similar model [2] determined the minimum sample size with a power of 80% (*p* ≤ 0.05).

The surgical procedure was carried out using the methods described by Reichert *et al.* [51]. Briefly, the right tibia was exposed by a 12 cm longitudinal incision. A broad dynamic compression plate (DCP) (10 mm × 4.5 mm holes, Synthes, West Chester, PA, USA) was temporarily fixed to the tibia. Parallel osteotomies were cut using an oscillating saw and a 3 cm middiaphyseal bone segment was excised. Care was taken to ensure the periosteum was completely removed in the defect zone and within 1 cm from the defect site on the proximal and distal fragment. The DCP was fixed proximally using four screws and the scaffold was inserted into the defect. The DCP was then affixed to the distal fragment using three screws, subjecting the scaffold to a press-fit condition. The wound was then closed in two layers using sutures.

### 4.6. Clinical Monitoring

Clinical X-rays (3.2 mAs; 65 kV, Philips Veradius, Amsterdam, NL, USA) were taken immediately after surgery and after six months healing time in two standard planes (anterior-posterior and medial-lateral) to determine the healing progress of the defect in the different experimental groups over time.

After six months the animals (which gained on average 20 to 30% weight) were euthanized by intravenous injection of 60 mg/kg sodium pentobarbital (Lethabarb, Virbac, Australia) and both the left (control tibiae) and the right tibiae (containing the scaffold) and 1 cm of surrounding tissue were explanted for subsequent mechanical testing, micro computed tomography and histological analysis.

### 4.7. Mechanical Testing

Mechanical testing was carried out at the same time post euthanasia for all samples and all samples were subjected to testing but only samples with enough mechanical strength to resist torque were suitable for testing. The fracture fixation plate was carefully removed from the experimental leg. Both ends of the tibia were embedded in stainless steel cups using 80 ml of dental acrylic (Paladur, Heraeus Kulzer, Hanau, Germany) and the bone was then mounted in an Instron 8874 biaxial testing machine (Instron, Norwood, MA, USA) according to published methods [41]. By leaving as much soft tissue as possible attached and by wrapping the bones with saline soaked gauze, bone samples were prevented from drying out. Next, the torsion test was conducted under a compressive load of 50 N and an angular velocity of 0.5 deg/s (right tibia counter clockwise, left tibia clockwise) until the bones failed and ultimate torque and torsional angle at failure were recorded. The torsional stiffness was calculated from the slope of the linear portion of the torque-angular displacement curves. Both ultimate torque and torsional stiffness of the experimental tibia were normalized against the values of the intact contralateral tibia.

### 4.8. Micro Computed Tomography

Samples were scanned in a micro computed tomography (microCT) scanner (μCT 40, Scanco Medical, Brüttisellen, Switzerland) with a voxel size of 36 μm. The X-ray tube was operated at 70kV and 114 μA. For further analysis, the scans were segmented using a threshold of 437.8 mg HA/ccm, a Gaussian filter width of 0.8 and filter support of 1.0 to identify mineralised tissue. The segmented scans were then analysed for bone volume and bone mineral density within the defect using the software supplied by the manufacturer. For bone volume, the regions of interest were determined as external callus formation (peripheral), bone formation within the scaffold (scaffold wall) and bone formation in the inner part of the scaffold (scaffold lumen). Defect bridging was defined as when new and continuous mineralised bone joined the proximal and distal regions of native bone.

### 4.9. Histological Sample Preparation

After micro CT scanning, tibial bone specimens were trimmed to five cm length (3 cm defect zone with 1 cm adjacent cortical bone proximally and distally) and fixed in 4% paraformaldehyde for one week before being transferred to 70% ethanol. For histological analysis, the mid-defect regions were sectioned in the transverse and sagittal plane. The sagittally sectioned samples were used for paraffin embedding. For processing decalcified samples into paraffin, bone samples were decalcified in 15% EDTA for 6–8 weeks at 4 °C. The samples were then dehydrated in increasing ethanol concentrations in a tissue processor (Excelsior ES, Thermo Scientific, Waltham, MA, USA), and embedded in paraffin. Five-micrometer sections were obtained using a rotary microtome (RM 2265, Leica, Wetzlar, Germany).

The mineralised bone samples to be cut on the sagittal plane were embedded in methylmethacrylate resin (Technovit 9100, Heraeus Kulzer, Hanau, Germany). Six-micrometer sections of the full length of the defect (5 cm long) were cut using a sledge microtome (Polycut S, Reichert technologies, Depew, NY, USA) and 40–50 µm sections were cut using an EXAKT cutting and grinding system (EXAKT, Norderstedt, Germany). Sections were cut at two thicknesses because the thicker ones preserve gross morphology more accurately and the thin sections reveal more cellular detail.

### 4.10. Von Kossa Staining of Mineralised Sections

Staining using the Von Kossa method was used to identify mineralised tissue which appears black and a MacNeil’s Tetrachrome stain showed cellular detail in the soft tissue. Resin sections (6 µm) were deplastified using acetone and rehydrated with serial concentrations of ethanol. Sections were immersed in silver nitrate solution (5%) for 5 min then developed with a sodium carbonate formaldehyde solution for 2 min. Slides were rinsed and immersed in Farmer’s Diminisher solution for 30 s. After further washing, slides were immersed in MacNeal’s Tetrachrome solution for 5 min with freshly prepared solution. The slides were then dehydrated and mounted using Eukitt mountant.

### 4.11. Goldner’s Trichrome Staining of Mineralised Sections

Resin sections (40–50 µm) were immersed in xylene followed by descending concentrations of ethanol. Samples were stained in Weigert’s Haematoxylin then washed. Slides were then stained in Ponceau-Fuchsin working solution (10 min) followed by acetic acid (1%). Slides were next immersed in Orange G solution (20 min) followed by acetic acid (1%). Finally, slides were immersed in Light Green solution (10 min) followed by acetic acid (1%). Slides were serially dehydrated and then coverslips were applied using Eukitt mountant.

### 4.12. Haematoxylin and Eosin Staining of Demineralised Sections

Paraffin sections were deparaffinized with xylene and rehydrated with decreasing concentrations of ethanol, before being stained with haematoxylin and eosin, dehydrated and mounted with Eukitt mountant.

### 4.13. Immunohistochemistry of Demineralised Sections

Paraffin sections were deparaffinised with xylene and rehydrated with decreasing concentrations of ethanol. Subsequently, sections were rinsed in distilled water and placed in 0.2 M tris(hydroxymethyl)aminomethane (Tris) buffer prepared at pH = 7.4. Endogenous peroxidase activity was blocked by incubating the sections in 3% H_2_O_2_ in Tris-HCl for 20 min. This was followed by three washes with Tris buffer (pH = 7.4) for 2 min each. Sections were incubated with Proteinase K (DAKO, Botany, Australia) for 20 min and subsequently incubated with 2% bovine serum albumin in DAKO antibody diluent in a humidified chamber at room temperature for 20 min to block nonspecific binding sites. 

Immunohistochemical staining was performed using a primary antibody specific to the markers of interest. The sections were incubated with the specific antibody in humidified chambers at 4 °C overnight (detailed below). Sections were then washed three times for 2 min with Tris buffer (pH = 7.4) and incubated with peroxidase labelled dextran polymer conjugated to goat anti-mouse and anti-rabbit immunoglobulins (DAKO EnVision+ Dual Link System Peroxidase, DAKO, Sydney, Australia) at room temperature in humidified chambers for 60 min. Colour was developed using a liquid 3,3-diaminobenzidine (DAB) based system (DAKO). Slides were stained with Haematoxylin to visualise cell nuclei then serially dehydrated and Eukitt coverslip mountant was used for coverslip mounting.

The selected antibodies were for Osteocalcin (OC4-30 used at a dilution of 1:250, abcam, Cambridge, MA, USA), Collagen 1 (COL-1 used at a dilution of 1:250, abcam, Cambridge, MA, USA), CD68 (MCA341GA at a dilution of 1:250, Serotec, Raleigh, NC, USA) and Von Willebrand Factor (IR527 supplied at a working concentration, DAKO, Botany, Australia).

### 4.14. Statistical analysis

Statistical analyses for the biomechanical results and for the micro computed tomography scans (micro CT) were carried out using a Mann-Whitney U test and p-values were adjusted using the Holm method [53]. All statistical analyses were carried out using the statistical software package R [54]. Results were considered significant at *p* < 0.05.

## 5. Conclusions 

The group administered with VEGF, PDGF and BMP-2 indicated greater blood vessel density in the mid-section of the scaffold. This apparent increased vascularisation had little effect on mineralised bone and bridging at the six-month timepoint. Further study would be required to quantitatively assess vasculature and ascertain if this increased vasculature results in a better long-term outcome. It appears that under the conditions evaluated, that the angiogenic GFs added showed no benefit to defect mineralisation and added significant costs to treatment groups. Further studies of controlled release BMPs alone are however a strong candidate for future study. By controlling the release of GFs we can more accurately mimic *in vivo* invironments. Biomimicry is the key to biologically relevant treatment approaches.

Although this study failed to determine the advantage of VEGF and PDGF administration during osteogenic regeneration, it did show that controlled release formations of BMP-2 can be effective when applied in a clinically relavent large animal model.

## Figures and Tables

**Table 1:** Three experimental groups, each with 5 animals. The microparticles deliver a calculated effective dose of 2 ng/mm^3^/day from the BMP-2 microparticles and 1 ng/mm^3^/day from the VEGF and PDGF microparticles.

Group	Growth Factor Loaded Microparticles
GF-free	Control microparticles (no growth factor)
Combination	VEGF (0.24 mg), PDGF (0.24 mg) and BMP-2 (1.12 mg)
BMP-2	BMP-2 only (1.12 mg)

**Table 2:** Triblock copolymer characteristics [22].

M_N_^a^	% Mole Lactide^a^	% Mole Glycolide^a^	M_N_^b^	M_W_^b^	PDIb
1706-1500-1706	71	29	2442	4022	1.65

adetermined by 1H NMR; bdetermined by GPC.

**Table 3:** Polymer formulations used to deliver the growth factors (VEGF, PDGF and BMP-2). Growth factor release

PLGA Lactide:Glycolide Ratio	PLGA-PEG-PLGA % w/w	Growth Factor
50:50	10	BMP-2
50:50	20	PDGF-BB
85:15	30	VEGF-165

## Supplementary Materials

The following are available online at www.mdpi.com/1996-1944/9/4/259/s1.

## Abbreviations

The following abbreviations are used in this manuscript:

BCA: Bicinchoninic acid
BMP: Bone morphogenetic protein
CD: Cluster of differentiation
CT: Computed tomography
DCP: Dynamic compression plate
GF: Growth factor
IHC: Immunihistochemistry
OC: Osteocalcin
P: P-value (test statistic)
PBS: Phospahte buffered saline
PCL: Polycaprolactone
PDGF: Platelet derived growth factror
PEG: Polyethylene glycol
PLGA: Poly(D,L-lactide-co-glycolide)
PRP: Platelet rich plasma
UV: Ultraviolet
VEGF: Vascular endothelial growth factor
vWF: Von Willebrand factor
