# Supplementary material for: Microparticles for Sustained Growth Factor Delivery in the Regeneration of Critically-Sized Segmental Tibial Bone Defects
Source: Materials (Basel). 2016 Mar 31;9(4):259. doi: 10.3390/ma9040259 (PMC5502923; doi:10.3390/ma9040259)
Supplement: Supplementary file 1 [file materials-09-00259-s001.pdf]

Supplementary Materials: Microparticles for Sustained Growth Factor Delivery in the Regeneration of Critically-Sized Segmental Tibial Bone Defects

Giles T. S. Kirby, Lisa J. White, Roland Steck, Arne Berner, Kristofor Bogoevski, Omar Qutachi, Brendan Jones, Siamak Saifzadeh, Dietmar W. Hutmacher, Kevin M. Shakesheff and Maria A. Woodruff

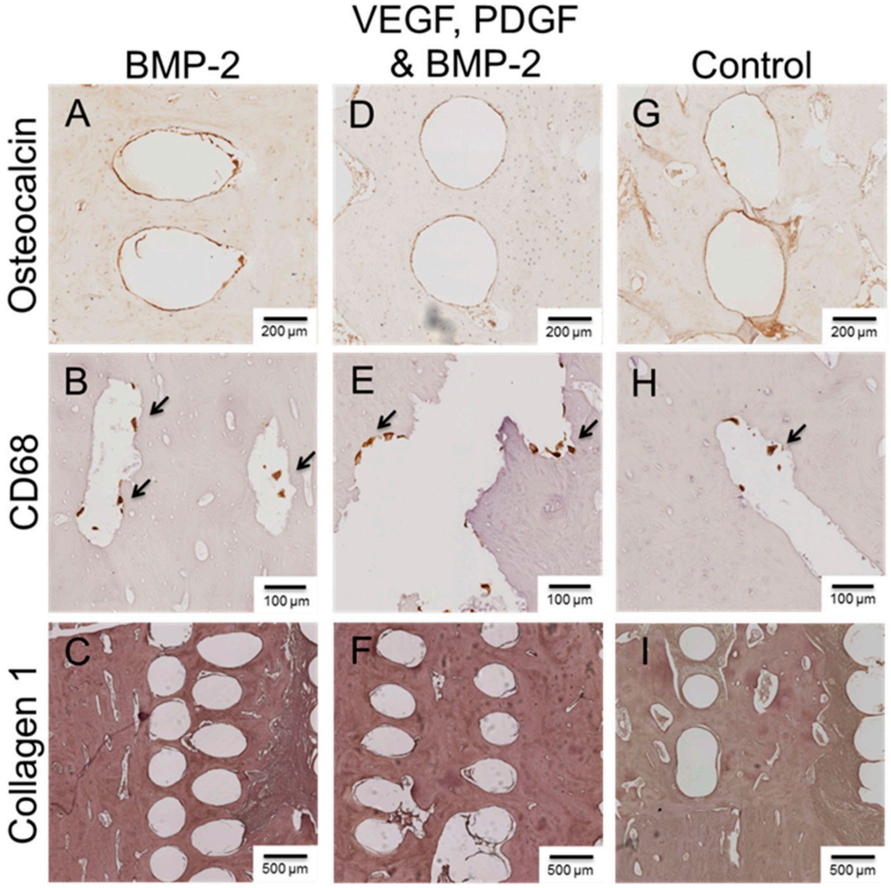

Figure S1. Representative images of proximal defect regions immunohistochemically stained for Osteocalcin, CD68 or Collagen 1. Defects were treated with controlled release BMP-2 (**A**–**C**), controlled release VEGF, PDGF; and BMP-2 (**D**–**F**) or control defects which were not administered with GF (**G**–**I**).

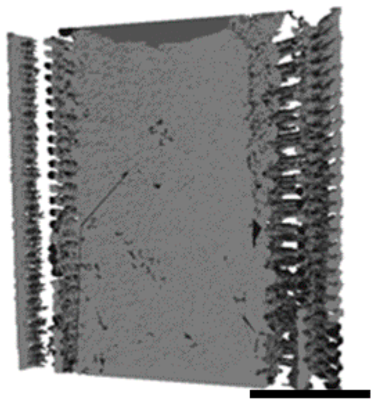

Figure S2. Micro CT image taken prior to implantation showing the PCL scaffold with PLGA micropaprticles applied into the central lumen. Scale bar is 1 cm.
